# 2196. Private Practice Dentists Answer the Dental Antibiotic Stewardship "Call to Action" with a Grassroots Initiative

**DOI:** 10.1093/ofid/ofad500.1818

**Published:** 2023-11-27

**Authors:** Debra A Goff, Julie E Mangino, Jason Stoner, Douglas Goff

**Affiliations:** The Ohio State University, Columbus, Ohio; The Ohio State University, Columbus, Ohio; Stoner Periodontic and Implant Specialists, Dublin, Ohio; Gilbert and Goff Prosthodontists, Columbus, Ohio

## Abstract

**Background:**

In the US, 90% of dentists work in private practice (PP). We aim to describe how infectious diseases (ID) antibiotic stewardship (AS) experts collaborated with dentists to provide dental AS education.

**Methods:**

Fifteen PP dentists completed a study conducted in 2021 by an ID-AS pharmacist and physician assessing their antibiotic use to identify AS opportunities. After learning dental AS from ID–AS experts, these dentists optimized and significantly decreased their antibiotic use. An AS “call to action” was launched in Sept. 2021 on how to expand our education to additional dentists to optimize antibiotic prescribing further. Dentists recommended ID-AS experts provide dental AS education to their long standing established local and national dental study club networks and other dental continuing education (CE) venues. Education focused on 5 areas: decreasing antibiotic use and durations, eliminating clindamycin and quinolones, incorporating shared decision making with patients, reviews of evidence based studies, and penicillin allergic patient management. Attendees were asked 3 questions prior to each lecture; 1. Have you heard of the term dental AS?; 2. Do you use clindamycin for penicillin allergic patients? 3. What is your standard duration of antibiotic therapy? Educational events were recorded through April 2023.

**Results:**

ID-AS experts provided AS education to 3742 PP dental providers, students/faculty over 20 months (Table 1). Prior to our lectures, most (90%) dentists were unfamiliar with dental AS; 88% prescribed clindamycin for penicillin allergies and 7 days were the most frequent duration for antibiotics.

**Figure:**
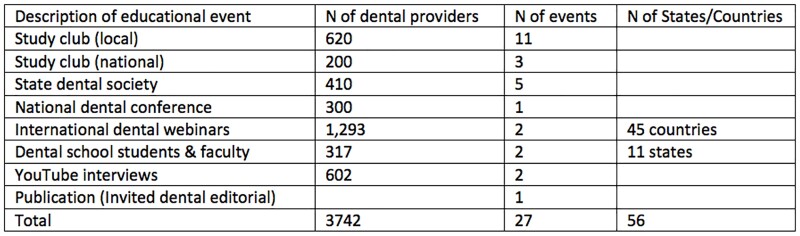

**Conclusion:**

PP dentists and faculty embraced AS education. Dental study clubs/CE forums provide excellent opportunities for ID-AS experts to engage and educate PP dentists. Dentists who attended AS lectures strongly support a 1-hour AS annual CE, similar to annual dental opioid CE required by many states.

**Disclosures:**

**All Authors**: No reported disclosures

